# SACRB-MAC: A High-Capacity MAC Protocol for Cognitive Radio Sensor Networks in Smart Grid

**DOI:** 10.3390/s16040464

**Published:** 2016-03-31

**Authors:** Zhutian Yang, Zhenguo Shi, Chunlin Jin

**Affiliations:** 1 School of Electronics and Information Engineering, Harbin Institute of Technology, Harbin 150001, China; shizhenguotvt@gmail.com; 2 State Grid Jilin Province Electric Power Research Institute, Changchun 130000, China; jinchunlin_grid@163.com

**Keywords:** smart grid, MAC protocol, cognitive radio sensor networks, capacity, spectrum aggregation

## Abstract

The Cognitive Radio Sensor Network (CRSN) is considered as a viable solution to enhance various aspects of the electric power grid and to realize a smart grid. However, several challenges for CRSNs are generated due to the harsh wireless environment in a smart grid. As a result, throughput and reliability become critical issues. On the other hand, the spectrum aggregation technique is expected to play an important role in CRSNs in a smart grid. By using spectrum aggregation, the throughput of CRSNs can be improved efficiently, so as to address the unique challenges of CRSNs in a smart grid. In this regard, we proposed Spectrum Aggregation Cognitive Receiver-Based MAC (SACRB-MAC), which employs the spectrum aggregation technique to improve the throughput performance of CRSNs in a smart grid. Moreover, SACRB-MAC is a receiver-based MAC protocol, which can provide a good reliability performance. Analytical and simulation results demonstrate that SACRB-MAC is a promising solution for CRSNs in a smart grid.

## 1. Introduction

The legacy electric power grids are facing numerous challenges, such as aging infrastructure, energy inefficiency, frequent transmission congestion and even failures. The next generation of electric grids, termed as smart grids, are expected to supply improved service with higher reliability, efficiency, agility and security [1,2,3], due to their capabilities of distributed computing, automated control and advanced bi-directional communications. Electricity providers, distributors and consumers would benefit from real-time awareness of operating environments, requirements and capabilities, since smart grids are capable of gathering information in real time from equipment in different areas and then making intelligent decisions to promote the efficiency and security of electric grids. For the successful operation of a smart grid, an integrated high performance, reliable, efficient, robust and secure communication network is critical [4,5].

Recently, the *Cognitive Radio Sensor Network*(CRSN) has gained much attention for electric power networks [6,7,8,9,10,11] and is expected to address the unique challenges of communications in a smart grid. In [6,7], the advantages of CRSNs are introduced, and the potential for the application in smart grids is analyzed. CRSNs can supply low cost, efficient and reliable operation in different parts of a smart grid, such as transmission towers, commercial buildings, distributed power plants, *etc*. The information gathered from sensors can be used for different applications, such as real-time fault detection, smart metering, automated demand response, and so on. Moreover, due to the capability of dynamic spectrum access, CRSNs can reduce congestion and excessive packet loss and thereby make transmissions more reliable. For the realization of CRSNs, literature [8] studies the connectivity of large-scale networks in fading environments under different routing mechanisms; literature [9] studies the coexistence and fairness, when several secondary networks share the same primary user resources; and [10] proposes a receiver-based MAC protocol for CRSNs in a smart grid (*i.e.*, Cognitive Receiver-Based (CRB)-MAC), which exploits the broadcast nature of the wireless medium to reduce retransmissions, so as to provide high energy efficiency and reliability. These research works make great contributions to successfully operating CRSNs in a smart grid. However, the high bandwidth requirements is a critical issue for the application of CRSNs in a smart grid, such as in *Advanced Metering Infrastructure* (AMI) networks [11,12], which are one of the key elements of a smart grid. Hence, the successful application of CRSN in a smart grid is dependent on the communication capabilities, which brings about enormous challenges for the reliability and throughput of CRSNs.

On the other hand, the spectrum aggregation [13] technique has been proposed to meet the increasing bandwidth demands and to overcome the problem of wide continuous spectrum band shortage in the current spectrum resource situation. Recently, a number of studies have been done on spectrum aggregation-based approaches for Cognitive Radio (CR) networks to improve throughput and spectrum efficiency (e.g., see [14,15,16,17,18,19,20,21,22]). Especially, in [20], a Spectrum-aware Clustering protocol for Energy-Efficient Multimedia routing (SCEEM) is proposed, in order to improve the routing efficiency and overcome the limitations of energy and spectrum, by using spectrum-aware clustering. In [21], a spectrum-aware and energy-efficient MAC protocol is proposed to improve the energy efficiency of CRSNs. By communications on a *Common Control Channel* (CCC), the sender and receiver can search the common available channels in subsets of the spectrum band, such that the energy consumption for spectrum sensing is deceased. In [22], Spectrum-aware and Cognitive Sensor Networks (SCSNs) are proposed for CRSN-based smart grid applications, which can overcome spatio-temporally varying spectrum characteristics and harsh environmental conditions. Therefore, there is a great potential of using spectrum aggregation for CRSNs in a smart grid. However, the utilization of spectrum aggregation-based CRSNs in a smart grid is still unexploited. It requires optimizations and enhancements for each layer of the protocol stack, especially the Medium Access Control (MAC) protocol.

Against this background, this paper aims to propose a high-capacity MAC protocol with novel modifications especially tailored for CRSNs in smart grid. In this regard, the SACRB-MAC (Spectrum Aggregation Cognitive Receiver-Based MAC) protocol is proposed. SACRB-MAC is a spectrum aggregation-based and receiver-based MAC protocol designed with special emphasis on reliability and capacity requirements of CRSNs in smart grid. It adopts spectrum aggregation technique to improve the available bandwidth and utilizes wireless medium broadcast nature to improve the reliability of CRSNs.

The rest of the paper is organized as follows. Section 2 describes the framework for SACRB-MAC including overview, system model and the protocol description. In Section 3, analytical models for different performance metrics are discussed, followed by performance evaluation in Section 4. Finally, the paper is concluded in Section 5.

## 2. SACRB-MAC Framework

### 2.1. Overview of SACRB-MAC

SACRB-MAC is a receiver-based MAC protocol, which is inherently different from *sender-based* MAC protocols. In sender-based MAC protocols, it is the sender that selects the receiver node from its forwarder list. Oppositely, in SACRB-MAC, no particular receiver node is determined. The sender node transmits the data packet by broadcasting such that all neighbor nodes in the communication range can receive the packet and compete to forward it.

SACRB-MAC is especially designed with emphasis on capacity. The channel aggregation with the highest capacity is selected during spectrum aggregation. The sender shares its available channel list over the *Common Control Channel* (CCC). Each receiver performs spectrum sensing and finds the common channels between the sender and itself. The channel aggregation with highest capacity of each receiver is determined and will be sent to the sender over CCC. To ensure the optimal spectrum aggregation scheme can be obtained by the sender, each receiver has to stay for a timer, which is dependent on the capacity potential of the receiver. The first spectrum aggregation scheme will be adopted by the sender for transmission.

A typical feature of any CR application is that nodes engaged in spectrum sensing will not transmit or receive data packets. Hence, the throughput performance of the network will be degraded. In SACRB-MAC, sensor nodes adopt the maximal transmission time subject to the interference constraint to improve the transmission efficiency. In addition, the CCC is a specified channel, which is always available and reliable.

### 2.2. System Model

**Figure 1:**
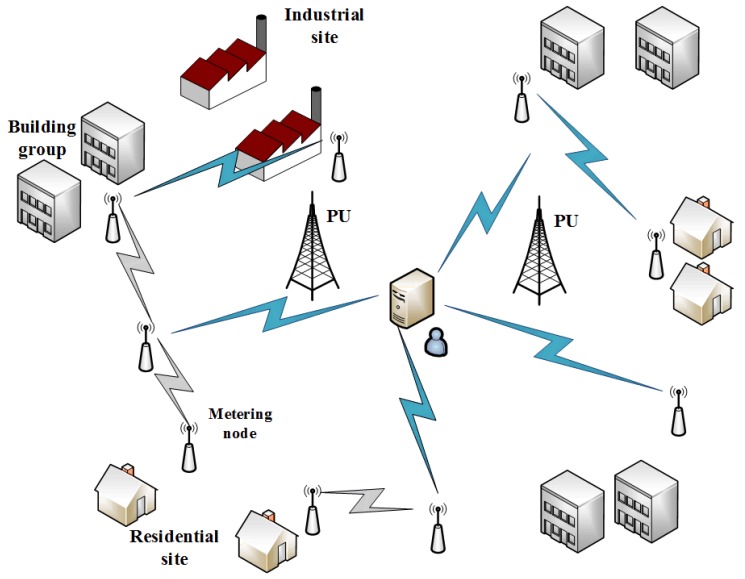
The schematic of AMI networks.

We consider the AMI network structured by using cognitive radio-equipped sensors, which is shown as Figure 1. In this AMI network, multiple cognitive radio-enabled meters (located at the customer premises) transmit customer information to a Meter Data Management System (MDMS), which acts as a control center for the management of meter data in order to be used by different applications (for AMI details, see [23]). We assume that only one secondary network (*i.e.*, the AMI network) exists in this region, and *N* stationary primary user (PU) transmitters (*N* spectrum band) exist with known locations and maximum coverage ranges. Moreover, *M* channels exist in each spectrum band. All CR meters adopt *Signal Interpretation before Fourier Transform* (SIFT) and *Frequency-Aware OFDM* (FA-OFDM) [24] techniques in radio transceivers and share a CCC for control information exchange, which is always available and reliable. Therefore, CR meters can aggregate channels from different spectrum bands and access these channels simultaneously for transmission [25].

Generally, the available spectrum for cognitive radio sensors is fragmented. The fragment size varies among different channels and bands. In order to detect these fragments, CR sensor nodes perform multiple channel spectrum sensing operation to the licensed spectrum bands. The status of channels are recorded in *Channel Status Table* (CST), which is given by:

(1) $$S={\left[\begin{array}{cccc} s_{11} & s_{12} & \cdots & s_{1M}\\ s_{21} & s_{22} & \cdots & s_{2M}\\ \vdots & \ddots & \vdots \\ s_{N1} & s_{N2} & \cdots & s_{NM} \end{array}\right]}_{N\times M},\;s_{j,i}\in \left\{0,1\right\}$$

where $s_{j,i}$ denotes the status of the *i*-th channel of the *j*-th band and $s_{j,i}=1$ indicates that the channel is available. The available channels for aggregation are selected based on CST. In addition, the CST is updated periodically based on the spectrum sensing results.

**Figure 2:**
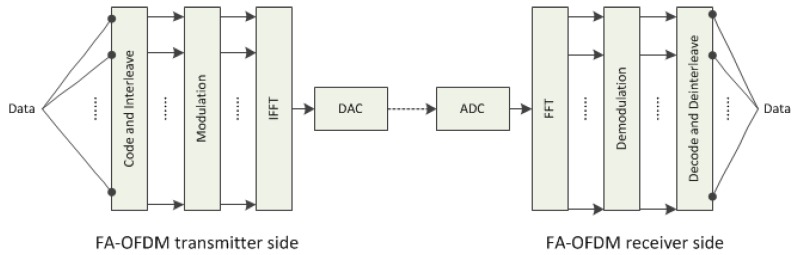
The schematic of FA-OFDM based PHY layer.

In FA-OFDM, different coding and modulation schemes are adopted in the OFDM sub-band, and the coding and modulation scheme of each sub-band is dependent on its signal-to-noise ratio. Accordingly, the data are split and carried by different carriers, which may adopt different transmit rates and power. The schematic of the FA-OFDM-based PHY layer is shown as Figure 2.

We also assume nonpreemptive CR node transmission when the wireless transceivers operates in transmission and reception mode. Even if the PU access the channel, the CR node will complete the current frame transmission and suspend the next frame data transmission. It is noted that PUs can access channels at any time instant, and their activities can be represented by a two-state independent and identically distributed random process, where the time of idle and busy periods is distributed with a mean of $\frac{1}{\mu_{OFF}^{i}}$ and $\frac{1}{\mu_{ON}^{i}}$, respectively. Let $S_{idle}^{j,i}$ denote the state that the $i^{th}$ channel in the $j^{th}$ is idle with probability $P_{idle}^{j,}$. Similarly, $S_{busy}^{j,i}$ denotes the state that PU is active in the $i^{th}$ channel (busy) in the $j^{th}$ with probability $P_{busy}^{j,i}=\frac{\mu_{OFF}^{i}}{\mu_{ON}^{i}+\mu_{OFF}^{i}}$, and $P_{idle}^{j,i}+P_{busy}^{j,i}=1$. The energy detection technique [26] is adopted here for PU detection, which is given by:

(2) $$Sensing\;Result=\left\{\begin{array}{cc} S_{busy}^{j,i} & \text{if}\;E^{j,i}\geq \sigma \\ S_{idle}^{j,i} & \text{if}\;E^{j,i}<\sigma \end{array}\right.$$

In spectrum sensing, the detection probability ($P_{d}$) and the false alarm probability ($P_{f}$) are two metrics. High detection probability ensures good protection to PUs and low false alarm probability ensures efficient utilization of channels. As description in [27], detection and false alarm probabilities for the $i^{th}$ channel in the $j^{th}$ band are given by:

(3) $$P_{d}^{j,i}=Pr\left\{E^{j,i}\geq \sigma |S_{busy}^{j,i}\right\}=Q\left(\frac{1}{\sqrt{2}}\frac{\sigma -2n_{i}\left(\gamma_{i}+1\right)}{\sqrt{4n_{i}\left(2\gamma_{i}+1\right)}}\right)$$

(4) $$P_{f}^{j,i}=Pr\left\{E^{j,i}\geq \sigma |S_{idle}^{j,i}\right\}=Q\left(\frac{1}{\sqrt{2}}\frac{\sigma -2n_{i}}{\sqrt{4n_{i}}}\right)$$

where Q(·) accounts for the Q function, which is the complementary error function, $\gamma_{i}$ denotes the primary signal-to-noise ratio and $n_{i}$ denotes the bandwidth time product of the *i*-th channel.

### 2.3. Protocol Description

#### 2.3.1. MAC Frame Structure

In SACRB-MAC, a MAC frame comprises two parts, *i.e.*, the spectrum sensing slot ($T_{ss}$) and the transmission slot ($T_{pt}$). In the spectrum sensing slot, the CR sensor checks the status of each channel in the spectrum aggregation list. The CR user will suspend the channels occupied by the PUs and access the available channels for transmission. We note that, in realistic conditions, PUs may be interfered with due to imperfect spectrum sensing. We use the *Interference Ratio* (IR) to quantify the interference. IR is defined as the expected ratio of PU transmission interrupted by CR users [10]. The IR for the *i*-th channel can be represented by:

(5) $$R_{I}^{j,i}=P_{busy}^{j,i}\left(1-P_{d}^{j,i}\right)+P_{idle}^{j,i}\left(1-P_{f}^{j,i}\right)+e^{-\mu T_{pr}}\left(P_{f}^{i}-P_{d}^{i}\right)$$

where $\mu =\text{max}(\mu_{ON}^{i},\mu_{OFF}^{i})$.

It is assumed that the CR nodes can use the transmission time excellently, such that the throughput of the CR network is maximized to an interference constraint, namely $R_{I}^{i}\leq R_{max}^{i}$, where $R_{max}^{i}$ denotes the maximum tolerable interference ratio on the *i*-th channel. Therefore, the transmission time on the *i*-th channel can be given by:

(6) $$T^{i}=\mu^{-1}\left[\ln P_{idle}^{j,i}-\ln\left(P_{idle}^{j,i}P_{d}^{\prime }+P_{busy}^{j,i}\left(1-P_{d}^{\prime }\right)-R_{max}^{i}\right)+\ln\left(2P_{d}^{\prime }-1\right)\right]$$

where $P_{d}^{\prime }$ is the detection probability at the tolerable SNR lower limit, which is specified by the regulator.

#### 2.3.2. Channel Selection Algorithm for Spectrum Aggregation

In channel selection for spectrum aggregation, the highest capacity that each receiver can provide is the key. Before responding to the sender, each receiver must stay for a duration $\Delta t_{y}$, where *y* denotes the index of receiver. $\Delta t_{y}$ is dependent on the highest capacity that receiver *y* can provide. Therefore, the receiver with better capacity potential can respond earlier so as to determine the channels to be aggregated.

Based on the Shannon’s Theorem, the capacity of the link between nodes *x* and *y* on the $i^{th}$ channel in $j^{th}$ band is as follows.

(7) $$C_{x,y}^{j,i}=B_{x,y}^{j,i}\log_{2}\left(1+SNR_{y}^{j,i}\right)\leq R_{D}$$

where $SNR_{y}^{j,i}$ and $B_{x,y}^{j,i}$ denote the received SNR at node *y* and the bandwidth of the $i^{th}$ channel in the $j^{th}$, respectively; $R_{D}$ is the minimum requested rate demand.

The received SNR of the $i^{th}$ channel in $j^{th}$ band at the node *y* can be given by

(8) $$SNR_{y}^{j,i}=\frac{P^{j,i}{\left|H_{x,y}\right|}^{2}}{\delta^{2}}$$

where $\delta^{2}$ denotes the power of noise, $P^{j,i}$ denotes the transmission power of *x* over the $i^{th}$ channel in the $j^{th}$ band, and $H_{x,y}$ denotes the channel coefficient, which can be given by

(9) Hx,y=Fx,yj,i11Lx,yj,iLx,y

where $F_{x,y}^{j,i}$ and $L_{x,y}^{j,i}$ denote the fading coefficient and pathloss of $i^{th}$ channel in the $j^{th}$, respectively. Therefore, the minimum transmission power for *x* over $i^{th}$ channel in the $j^{th}$ band can be given by:

(10) $$P_{min\_x}^{j,i}=\frac{\left(2^{\frac{R_{D}}{B_{x,y}^{j,i}}}-1\right)\delta^{2}}{\left|H_{x,y}^{2}\right|}$$

As per [25], the maximal capacity of the link can be achieved by solving the optimization problem.

(11) $$\begin{array}{cc} P: & \;\text{max}\;\sum_{j=1}^{N}\sum_{i=1}^{M}C_{x,y}^{j,i}s_{j,i}\\ s.t.\; & \left(a\right)\;P_{x}^{j,i}\leq P_{m}^{j,i},\;\;\forall \;j\in N,\forall \;i\in M\\ & \left(b\right)\;\sum_{j=1}^{N}\sum_{i=1}^{M}P_{x}^{j,i}s_{j,i}\leq P_{max}\\ & \left(c\right)\;\sum_{j=1}^{N}\sum_{i=1}^{M}s_{j,i}\leq \epsilon \\ & \left(d\right)\;\sum_{j=1}^{N}\sum_{i=1}^{M}C_{x,y}^{j,i}s_{j,i}\;\geq R_{D}\;\\ & \left(e\right)\;s_{i,j}\in S,\;\;\forall \;j\in N,\forall \;i\in M \end{array}$$

where $P_{m}^{j,i}$ denotes the maximum transmission power on the $i^{th}$ channel of the $j^{th}$ band, *ϵ* denotes the maximum number of channels aggregated due to the hardware constraints, and $P_{max}$ denotes the maximum of total transmission power for a node.

By solving the optimization problem, the highest capacity that the receiver can provide is obtained. Therefore, the timer $\Delta t_{y}$ for the receiver *y* before responding the preamble of the sender node *x* is given by:

(12) $$\Delta t_{y}=\omega {\left(C_{max\_x,y}\right)}^{-1}+t_{0}$$

where $C_{max\_x,y}$ denotes the maximal capacity of the link between node *x* and *y* using spectrum aggregation, and *ω* and $t_{0}$ are two constants.

#### 2.3.3. Next-Hop Competition Mechanism

In SACRB-MAC, nodes have no forwarder lists. The sender does not select a particular receiver. It is the receiver nodes that decide the next hop node, which is similar with receiver-based MAC protocols (such as CRB-MAC in literature [10]).It is assumed that the sender node *S* has data packets to transmit. First, *S* implements spectrum sensing (with duration $T_{ss}$) to detect PU activities. If all of channels are busy ($S_{busy}^{i}$), the node *S* will turn to sleep mode and waits for available channels. The spectrum sensing operation will be repeated after the duration of checking interval ($T_{CI}$). If available channels ($S_{idle}^{i}$) are detected, *S* will start broadcasting the preamble over CCC and the test packet over all available channels for receiver SNR determination. The preamble is composed of multiple micro-frames and lasts for $T_{pr}$. Each micro-frame lasts for $T_{m}$ and contains essential information for data transmission and spectrum aggregation, such as identification information and available channel list. All the one-hop neighbor nodes of *S* will detect the micro-frames and test packet. Then, the data packet can be identified based on the identification information, and received SNR for each available channel can be calculated.

We note that all nodes in the communication range of *S* are receiver candidates. However, only the nodes, which can access all the selected channels, will receive the data packet and are termed as *valid receivers*. For example, five neighboring nodes of *S* (*i.e.*, nodes *A*, *B*, *C*, *D* and *E*) are eligible to forward the data. They wake up and receive the preamble on CCC. Based on the available channel information of the sender and spectrum sensing result, each node can calculate out the the highest capacity of the link between the sender and itself. It is assumed that node *A* responds first. After the duration $\Delta t_{A}$, node *A* gives the response preamble to the sender *S* on CCC, in which the channel list for spectrum aggregation is included. Based on the response preamble, *S* can split data packet on the selected channels and broadcast the packet in the aggregated band.

Moreover, all of other neighbor nodes (*i.e.*, *B*, *C*, *D*, *E*) will check the selected channels. If the channels are available, these nodes will also receive the data from *S*. If the received data packet is detected to be erroneous, it will be simply discarded. The receiver which accomplishes the data packet receiving first and has available channels for transmission will send the preamble in CCC and apply to forward the data. Moreover, if receivers do not begin the preamble transmission on CCC in $t_{m}$, this forwarding will be discarded.

In addition, *S* will retransmit the data packet if none of the nodes within its transmission range transmits the response preamble in the contention window. This can be found by *S* by performing sensing operation before ending the contention window ($T_{CW}$), where $T_{CW}$ is dependent on the transmission radius of *S*. The algorithm of response-based next hop competition mechanism is shown in Algorithm 1.
**Algorithm 1:** Response-based Forwarder Competition Mechanism
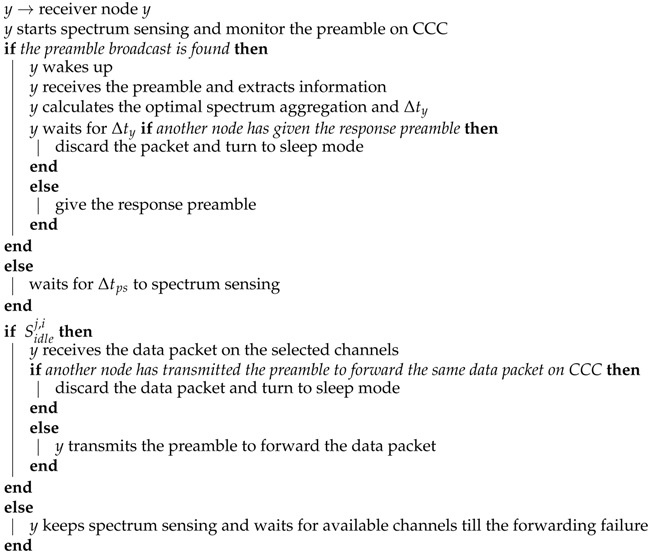


## 3. Analytical Model

The CR nodes can only access the licensed channel when no PU activities are found. Therefore, each CR user has a probability to access some licensed channel. Let $P_{ac}^{j,i}$ denote the probability for CR users to access the $i^{th}$ channel in the jth band, which can be given by:

(13) $$P_{ac}^{j,i}=P_{busy}^{j,i}\left(1-P_{d}^{i}\right)+P_{idle}^{j,i}\left(1-P_{f}^{i}\right),\;\;i\in S$$

In the SACRB-MAC, the probability of single hop transmission failure over the $i^{th}$ channel in the $j^{th}$ band is given by:

(14) $$P_{fail}^{j,i}=P_{ac}^{j,i}\left[1-{\left(1-p_{b}\right)}^{d+m}\right]$$

where *d* and *m* denote the sizes of data frame and micro-frame in bits, respectively; $p_{b}$ is the bit error probability.

Reliability is an important property of a network and usually evaluated in terms of *Packet Delivery Ratio* (PDR) [10], which is defined as the fraction of the numbers of packets received and packets sent. Analytically, the reliability for SACRB-MAC (with $N_{R}$ valid receivers) on the selected channel set SC is given by:

(15) $$\mathrm{PDR}=1-{\left[1-\prod_{s=1}^{S}\left(1-P_{fail}^{s}\right)\right]}^{N_{R}\left(\chi +1\right)}$$

where *S* denotes the number of selected channels, and $P_{fail}^{s}$ denotes the probability of single hop transmission failure over the $s^{th}$ selected channel.

The single hop delay for SACRB-MAC over the selected channels is given by:

(16) $$D=\chi \cdot \left(T_{pr}+T_{d}+T_{CW}\right)+\chi_{ss}\cdot T_{ss}+\Delta t_{y}+T_{CI}\cdot \left(\chi_{ss}-1\right)$$

where $T_{pr}$ denotes the duration of the preamble frame, $T_{d}$ denotes the duration of data frame, $\chi_{SS}$ denotes the number of spectrum sensing events, and *χ* denotes the retransmission count, which is given by:

(17) $$\chi =\sum_{k=1}^{K}{k\cdot P_{k}=\left(1-\prod_{s=1}^{S}\left(1-P_{fail}^{s}\right)\right)}^{k\cdot N_{R}}\left[1-{\left(1-\prod_{s=1}^{S}\left(1-P_{fail}^{s}\right)\right)}^{N_{R}}\right]\;$$

where $P_{k}$ denotes the probability that the transmission is successful after *k* retransmissions, and *K* denotes the maximum number of retransmissions. For a multi-hop scenario, the end-to-end delay over *Q* hops is given by $\sum_{q=1}^{Q}D$.

The potential bandwidth of the $i^{th}$ channel in the $j^{th}$ band can be represented by:

(18) $$W_{x,y}^{j,i}=P_{ac}^{j,i}B_{x,y}^{j,i}$$

Therefore, the total capacity of the link between *x* and *y* is given by:

(19) $$C_{x,y}=\sum_{s=1}^{S}P_{ac}^{s}\cdot C_{x,y}^{s}$$

where $C_{x,y}^{s}$ denotes the capacity of the $s^{th}$ selected channel, and $P_{ac}^{s}$ denotes the probability to access the $s^{th}$ selected channel.

## 4. Results and Discussion

**Figure 3:**
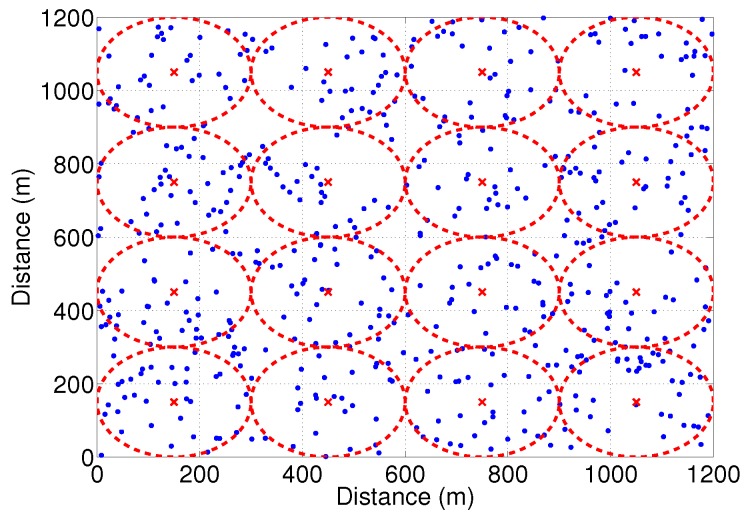
Sample simulated topology with Poisson distributed nodes (density = 400 nodes per square kilometers). The filled squares and dotted circles represent the location and coverage area of PU transmitters, respectively.

In this section, the performances of SACRB-MAC on a single hop and multiple hops in smart grid AMI networks will be evaluated. A MATLAB based simulation is performed to verify the analytical models. Specifically, a square region of side 1200 m with 16 PU existing is considered. We assume that the cognitive radio sensors with transmission the radius of 150 meter are Poisson distributed in the whole region with a mean density, which is shown in Figure 3. Without loss of generality, we assume that Routing Protocol for Low Power and Lossy Networks (RPL) is adopted at the Network layer. The parameters of the simulation are as shown in Table 1. In addition, we also perform a receiver-based MAC protocol (CRB-MAC) and a sender-based MAC protocol (1-hopMAC [28]) under the cognitive radio environment (CSB-MAC) for comparison.

**Figure 4:**
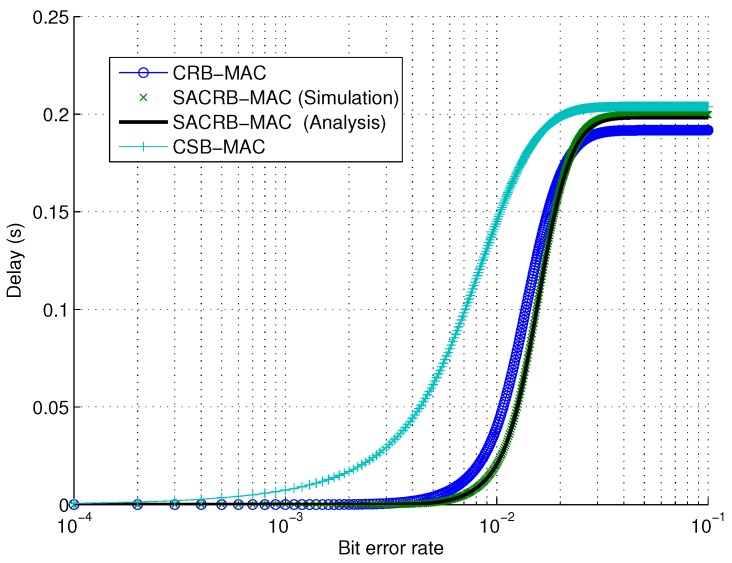
Single hop delay performance against bit error rate.

First, we evaluate the performance of delay. The single hop delay against bit error rate (BER) is evaluated in Figure 4. The two receiver-based protocols have better performance. This is because that the delay performance is mainly dependent on the number of retransmissions. Due to the receiver-based nature, SACRB-MAC and CRB-MAC have fewer retransmissions than CSB-MAC. Moreover, the delay performance has a saturation point when the retransmission count reaches the maximum.

**Figure 5:**
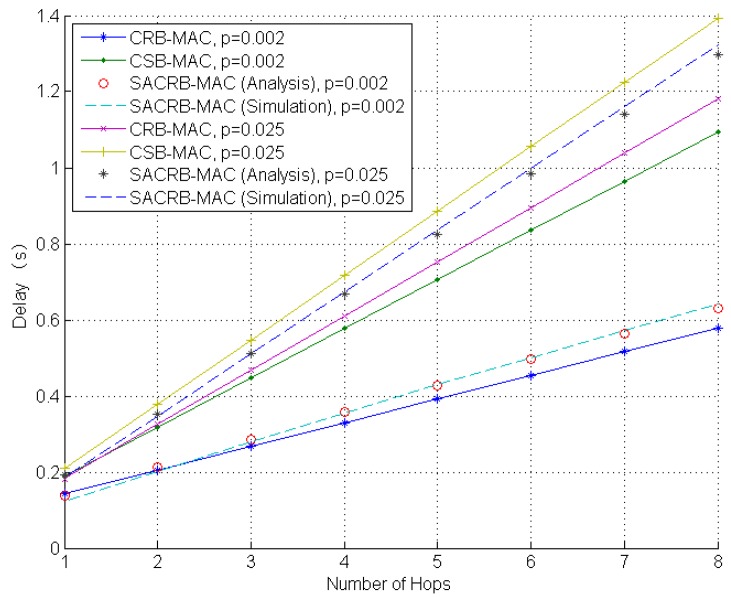
Multi-hop delay performance against number of hops.

In Figure 5, the performance of multi-hop delay is evaluated. We note that the end-to-end delay increases linearly as the number of hops increases. CRB-MAC and SACRB-MAC outperform CSB-MAC in the performance of end-to-end delay (in both low and high BER scenarios) due to fewer retransmissions. Besides, the performance of SACRB-MAC is near to that of CRB-MAC. In addition, the simulation results agree with the analytical ones.

**Figure 6:**
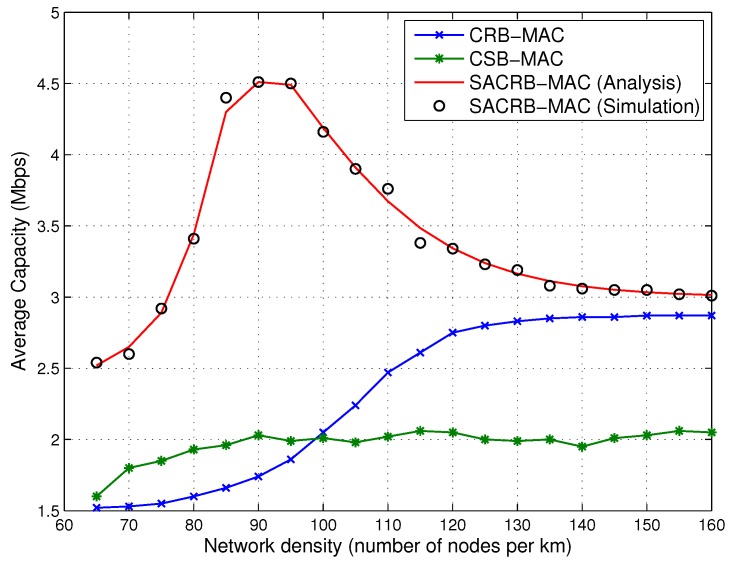
Average capacity performance against network density.

Next, we evaluate the capacity performance. The analytical and simulation results for the average capacity against network density are given in Figure 6. It is noted that the average capacity performance of SACRB-MAC initially increases and then decreases as the network density grows. This is because the probability of finding a receiver node with higher capacity grows with the network density. However, the probability of the preamble packet collision on the CCC increases as the network density increases. In high network density environments, the collision may cause average capacity degradation. In comparison, SACRB-MAC outperforms CRB-MAC and CSB-MAC obviously, because of the adoption of the spectrum aggregation technique. Besides, the simulation result agrees with the analytical one.

**Figure 7:**
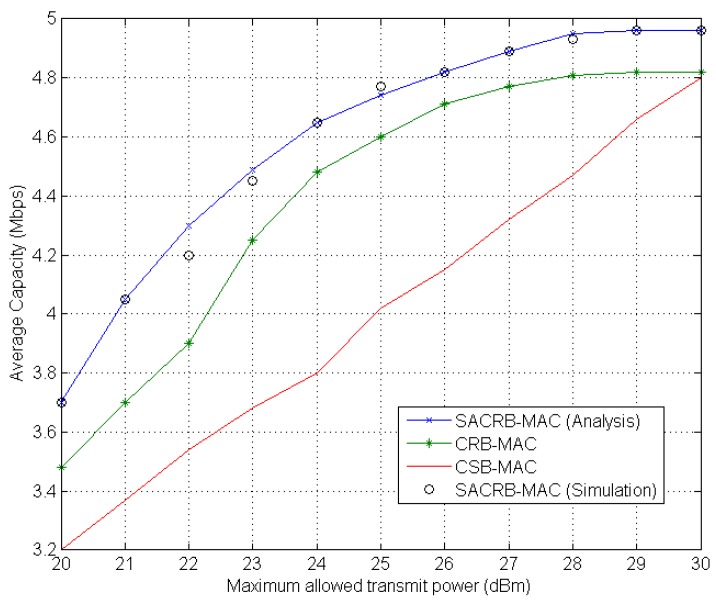
Average capacity performance against the maximum allowed transmit power of each channel.

We evaluate the average capacity against the maximum transmitting power allowed in each channel. The analytical and simulation results are given in Figure 7. It is assumed that the maximum transmitting power allowed in each channel is fixed, which ranges between 20 and 30 dBm, and the maximum power constraint of each cognitive radio sensor node is 30 dBm. The average capacity increases as the maximum allowed transmitting power increases. However, the average capacity reaches the saturation point when the total transmission power reaches the maximum power constraint.

**Figure 8:**
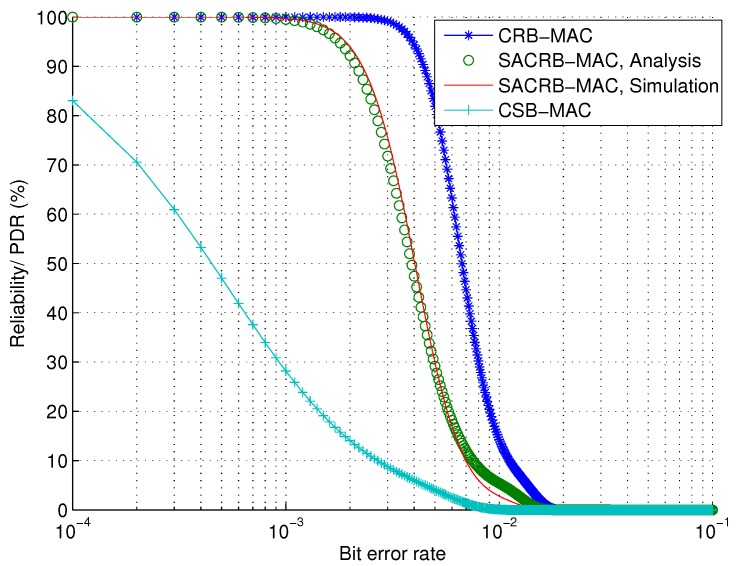
Reliability performance against bit error rate in terms of PDR.

Last, but not the least, we evaluate the reliability performance. The reliability in terms of PDR against the bit error rate is shown in Figure 8. SACRB-MAC and CRB-MAC have better reliability performances. This is because more receivers are participating in the data forwarding process, due to the receiver-based mechanism. Therefore, we can draw the conclusion that SACRB-MAC shows resiliency to channel quality variations and provides high reliability.

## 5. Conclusions

In this paper, we have proposed SACRB-MAC, which is a spectrum aggregation based MAC protocol. SACRB-MAC employs spectrum aggregation technique to improve the throughput of CRSNs in smart grid. Besides, SACRB-MAC is a receiver-based MAC protocol, which exploits the broadcast nature of wireless medium to improve reliability of CRSNs. Hence, SACRB-MAC has the potential to address the unique challenges of CRSNs in smart grid. Analytical and simulation results demonstrate that under smart grid AMI network environments, SACRB-MAC can have a high-capacity and reliable performance. Therefore, SACRB-MAC provides a promising solution for CRSNs in realizing the vision of smart grid. The coexistence and fairness for several secondary networks sharing the same primary user resources will be the focus of our future work.

## Figures and Tables

**Table 1:** Simulation Configuration Parameters.

Parameter	Value
Detection probability threshold ($P_{d}^{\prime }$)	0.9
Probability of false alarm ($P_{f}$)	0.1
Channel bandwidth	2 MHz
PU received SNR (*γ*)	−15 dB
Busy state parameter of PU ($\mu_{ON}$)	2
Idle state parameter of PU ($\mu_{OFF}$)	3
Maximum Interference Ratio ($IR_{max}$)	0.25
Spectrum sensing duration ($T_{s}$)	20 ms
CR node transmission range	150 m
Maximum transmitting power of each node ($P_{max}$)	30 dBm
Maximum allowed transmitting power of channels ($P_{j,i}^{m}$)	20–30 dBm
Checking interval ($T_{CI}$)	144 ms
Preamble length ($T_{pr}$)	144 ms
Transmission time of a data packet ($T_{d}$)	4 ms
Transmission time of one micro-frame ($T_{m}$)	40 μs
Transition time from sleep mode to active mode (*τ*)	88.4 μs
